# Assessing the Impact of Single-Cell Stimulation on Local Networks in Rat Barrel Cortex—A Feasibility Study

**DOI:** 10.3390/ijms20102604

**Published:** 2019-05-27

**Authors:** Beate Knauer, Maik C. Stüttgen

**Affiliations:** 1 University Medical Center of the Johannes Gutenberg University Mainz, Institute of Pathophysiology, 55128 Mainz, Germany; 2 Focus Program Translational Neurosciences, University Medical Center of the Johannes Gutenberg University Mainz, 55128 Mainz, Germany

**Keywords:** barrel cortex, nanostimulation, juxtacellular, in vivo

## Abstract

In contrast to the long-standing notion that the role of individual neurons in population activity is vanishingly small, recent studies have shown that electrical activation of only a single cortical neuron can have measurable effects on global brain state, movement, and perception. Although highly important for understanding how neuronal activity in cortex is orchestrated, the cellular and network mechanisms underlying this phenomenon are unresolved. Here, we first briefly review the current state of knowledge regarding the phenomenon of single-cell induced network modulation and discuss possible underpinnings. Secondly, we show proof of principle for an experimental approach to elucidate the mechanisms of single-cell induced changes in cortical activity. The setup allows simultaneous recordings of the spiking activity of multiple neurons across all layers of the cortex using a multi-electrode array, while manipulating the activity of one individual neuron in close proximity to the array. We demonstrate that single cells can be recorded and stimulated reliably for hundreds of trials, conferring high statistical power even for expectedly small effects of single-neuron spiking on network activity. Preliminary results suggest that single-cell stimulation on average decreases the firing rate of local network units. We expect that characterization of the spatiotemporal spread of single-cell evoked activity across layers and columns will yield novel insights into intracortical processing.

## 1. Introduction

Despite decades of research, it remains essentially a mystery how neuronal networks process information [1]. In view of the huge number of neurons in vertebrate brains, many researchers assume that “as in any good democracy, individual neurons count for little; it is population activity that matters […] As single neurons are not very informative, to obtain accurate information about sensory or motor variables some sort of population averaging must be performed” [2]. Indeed, responses of individual neurons in sensory cortex in response to repeated presentations of physically identical stimuli displays high trial-by-trial variability [3], which can in principle severely hamper the decoding of information by downstream neurons. For example, the vast majority of neurons in the whisker area of primary somatosensory cortex (“barrel cortex” [4]) provide relatively little information on both the identity of a stimulated whisker and the frequency at which it is stimulated [5]. Unsurprisingly, neuronal ensembles consistently surpass single cells in terms of conveyed information. For example, small ensembles of neurons in rat somatosensory cortex outperform individual neurons by a factor of seven in determining the identity of a single stimulated whisker [6].

These general findings notwithstanding, other studies suggest that individual neurons can, under certain circumstances, confer a highly precise representation of sensory information. For example, Bair and Koch [7] demonstrated that trial-by-trial variability of neuronal responses in macaque extrastriate cortex is markedly reduced when dynamic visual stimuli rather than static displays are presented. Furthermore, about 50% of neurons in primate middle temporal visual area provide as-good or better discriminability of random-dot kinematograms than the highly trained macaques themselves [8]. Lastly, small pools of only a handful of neurons are sufficient to explain psychophysical detection performance for brief single-whisker deflections close to perceptual threshold [9,10]. Importantly, theoretical work suggests that the information content of neuronal ensembles saturates at relatively small pool sizes due to correlated firing of individual neurons [11]. From a computational perspective, sparse representations—i.e., ones in which only a small percentage of neurons is active at any given time—confer several advantages over more “dense” representations [12]. Additionally, it has been argued that sparse representations in cortex are demanded by limited availability of energy resources [13,14].

Of course, even when high stimulus coding precision of single neurons is observed, this does not necessitate that subsequent processing stages can benefit from this precision, especially when a much larger fraction of neurons provides much noisier signals. Therefore, a more direct test of the role of the single cells in neuronal computation is to electrically stimulate a small, well-defined ensemble of neurons and observe the effects on network activity [15] or behavior [16,17]. However, electrical microstimulation activates an unknown number of neurons scattered across a large area of cortex; thus, neither the number of activated cells nor the number of induced action potentials nor the effect of stimulating particular cells types can be assessed [18].

Overcoming these limitations, Brecht et al. [19] stimulated single neurons in infragranular layers of rat primary motor cortex and found that single-cell stimulation of pyramidal neurons is capable of producing a behavioral output, i.e., bursts of low-amplitude rhythmic whisking. The latency and amplitude of the movement depended on the number and the frequency of evoked spikes, respectively. The main conclusion is that, in vibrissa motor cortex, activity modulation of single neurons in awake animals indeed can have an effect on ongoing behavior. Stimulating single neurons in the lateral facial nucleus which directly project to the whisker musculature revealed that even a single spike is sufficient to evoke detectable whisker movements [20].

This approach of ‘reverse physiology’ was taken further by stimulating single cells in rat barrel cortex while the animal was engaged in a behavioral detection task [21]. Instead of whole-cell recordings, the authors adopted and modified the juxtacellular labeling method originally described by Pinault [22,23]. With this method (“nanostimulation”), the authors demonstrated that the injection of ~14 action potentials in a single cortical neuron can lead to a perceptually detectable event in about 5% of stimulation trials. Beyond being single-cell specific, a particularly interesting feature of nanostimulation is that it allows the labeling and, thereby, subsequent recovery of the stimulated cell. Taking advantage of this feature, Doron et al. [24] and Tanke et al. [25] showed that the behavioral effect of nanostimulation differed widely across cell types. Whereas stimulation of putative excitatory cells led only to weak biases, stimulation of putative inhibitory interneurons resulted in much stronger effects on sensation. Thus, the method of nanostimulation allows to relate the morphology, location, and firing pattern of a single neuron to complex perceptual events, assessed in operantly conditioned animals.

Even in small mammals such as rats and mice, the cerebral cortex consists of millions of neurons [26]. The marked impact that single neurons can have on network activity raises the important question how the brain, as a whole, can function reliably when at least a few of its millions of constituent elements are able to profoundly influence processing. In order to elucidate the phenomenon of single-cell induced network modulation, we set up an experimental arrangement allowing us to continuously observe network activity (as indexed by local field potentials and extracellular action potentials) via multi-electrode arrays while manipulating the activity of a single neuron (via nanostimulation) in the vicinity of these electrodes. In the next section, we provide a detailed description of the method, discuss pitfalls, and present some preliminary results suggesting that single-cell activation yields measurable effects on network activity in this configuration.

## 2. Results and Discussion

In the following, we will first describe components of the basic setup, and then move on to establishment of a stable recording configuration. Lastly, we present some preliminary results as proof of principle.

In short, the basic setup consisted of a head-holder for the rat, one micromanipulator to position the glass pipette for juxtacellular recording and stimulation, another micromanipulator to position a multi-electrode array (single- or multi-shank silicon probes), and a whisker stimulator. Establishing the aforementioned technique bears multiple challenges, including the achievement of a short (~300 µm) distance between the multi-electrode array and glass pipette.

### 2.1. The Basic Setup

**Figure 1:**
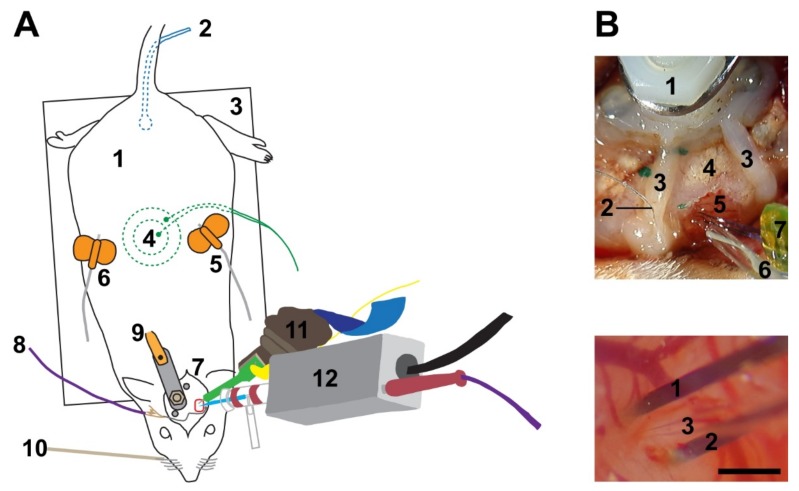
Experimental set up. (**A**) (1) Anesthetized rat. (2) Rectal probe to monitor body temperature. (3) Heating pad to control body temperature. (4) Piezo-ceramic element to record breathing rate. (5 + 6) Butterfly needles to apply anesthetics (5) and liquid supplements (6) subcutaneously. (7) Surgical site, including a stereotactically guided trepanation (red rectangle) above the barrel cortex. (8) Grounding wire (chlorided silver ball) in close proximity to the craniotomy. (9) Head post firmly fixed to the skull of the contralateral hemisphere. (10) Piezo-electric element for whisker stimulation. (11) Connector with a 32-channel silicon probe to record local network activity. (12) Head stage with a 3-8 MΩ glass pipette to record and stimulate single cells. (**B**) Top: overview of the surgical site. (1) Head post for fixation. (2) Grounding wire. (3) Dental cement wall to facilitate liquid retention. (4) Skull. (5) Brain. (6) Glass pipette with chlorided silver wire inside. (7) Silicon probe with two shanks. Bottom: close up on a pipette penetration in close proximity to an inserted double-shank silicon probe. (1) Left and (2) right shank of the silicon probe. (3) Glass pipette. Scale bar: 500 µm.

As the connection probability between neighboring cortical neurons falls off steeply with distance [27,28,29], and because we expect that the effect of single-cell stimulation is confined to the immediate vicinity of the stimulated cell, we aimed to bring the tip of the glass pipette within a few hundred µm of the silicon probe (Figure 1). To that end, both the pipette and the silicon probe are attached to rotatable and inclinable motorized micromanipulators. 

Rats were anesthetized either with urethane or a three-component anesthetic mixture containing fentanyl, medetomidine, and midazolam (3-C, see Methods for details). During initial preparation in a stereotaxic frame, a head post was implanted over the non-experimental side and caudal part of the skull, and a craniotomy was created over the barrel cortex. To gain easier access to the head and to be able to stimulate whiskers mechanically, the animals were then freed from the stereotaxic frame, and the head was fixed to a custom-built contraption (Figure 1A). The dura mater was incised under visual control and the approximately 33° angled silicon probe was slowly (2 µm/s) lowered into the cortex until only the most superficial electrode contact was barely visible at the surface of the brain. Thereafter, the tip of the glass pipette was positioned in close vicinity to the probe. The head stage with the pipette was tilted 30° to 35° to allow a penetration angle near perpendicular to the cortical surface (Figure 1B) in seven animals. In the remaining animals the pipette was advanced vertically.

### 2.2. Juxtacellular Stimulation

After the probe was in place, the glass pipette was advanced in 2 µm steps from superficial to deep cortical layers. Neurons were detected by an increase of the voltage deflection in response to a 200 ms, 1 nA square current pulse (“search pulse”), indicating an increase in resistance at the tip of the pipette. If the matter in front of the pipette responded to current injection with action potentials of an amplitude greater than 1.5 mV, the experimental protocol was commenced. Aside from spontaneously active neurons, this approach allowed the sampling of neurons exhibiting extremely low or even no spontaneous activity. The experimental electrical stimulus was a 200 ms square pulse with an additional rise and fall time of 10 ms, set to a current amplitude sufficient to elicit firing rates discernably above baseline level, and presented every 2 or 5 s. A more detailed description of nanostimulation can be found elsewhere [30,31].

#### 2.2.1. Respiratory and Pulse-Related Artifacts

**Figure 2:**
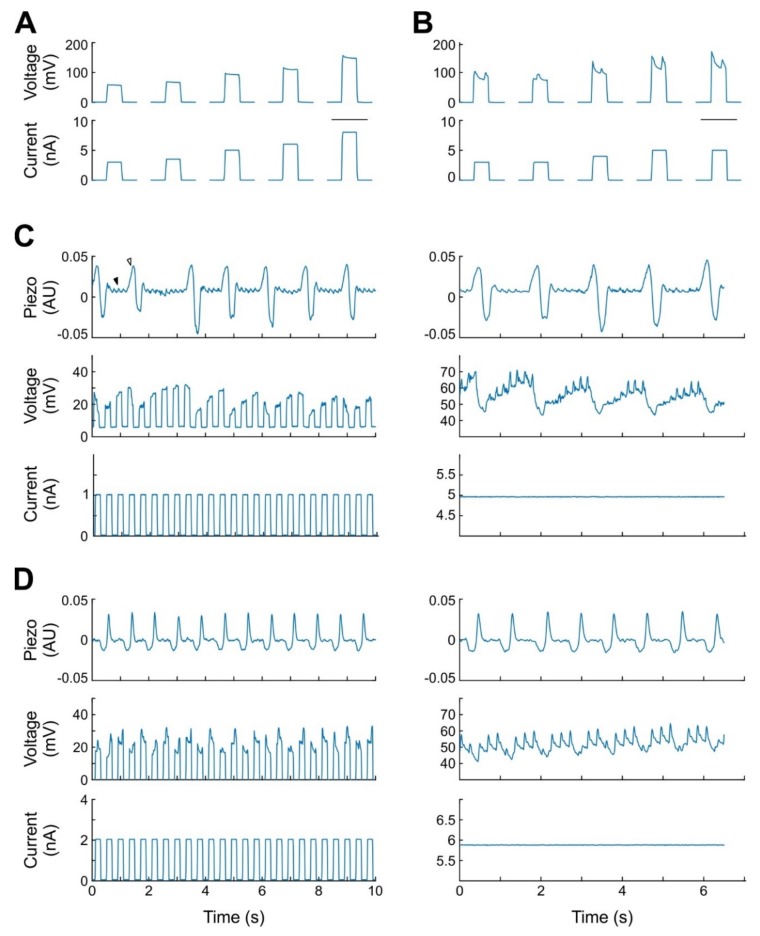
Respiration- and pulse-related interfering signals. (**A**) Juxtaposed stimulation and recording without interfering signals. Inter-stimulus intervals between the nano pulses omitted. Scale bar: 500 ms. (**B**) Same as A, but demonstrating interfering signals. (**C**) Continuous juxtaposed stimulation and recording from an animal anesthetized with 3-C. Top: representative respiratory (open arrow head) and pulse (closed arrow head) signal picked up from a mechanoelectric transducer underneath the chest. Left: application of a 200 ms square current pulse. Strong modulation of the potential by each breath. Right: elevated holding current. Strong modulation both by the breathing and the heartbeat. (**D**) Same as C, but from an animal anesthetized with Urethane. Top: representative respiratory signal. Weaker breathing, but stronger heart beat related signal in the recording of the voltage at the pipette.

Reliable induction of potentials with a steady plateau phase in the juxtacellular configuration (Figure 2A) is of paramount importance for the experiment. In our initial attempts at establishing the protocol, we noticed interfering signals at the glass pipette (Figure 2B), which rendered reliable stimulation of neurons in the juxtacellular configuration unfeasible. These signals, presented as voltage fluctuations, which emerged upon the increase of resistance at the pipette, were usually absent in the absence of current injection but aggravated at higher potentials (i.e., during the plateau of a square pulse or with elevated holding current). Due to the long inter-stimulus intervals (2 or 5 s) no regularity in the interfering signal could be detected. The correlation of the signals with respiration (Figure 2C) and pulse (Figure 2D) could only be determined by more frequent or continuous current application and concomitant recording of respiration- and pulse-signals from the chest of the animal. The interfering signals could not be reduced sufficiently by altering the shape of the pipette shank or tip, different placement of the grounding wire, reducing the size of the craniotomy, covering the brain with agar, systemic application of atropine, or repositioning the animal’s body.

All of the 11 animals anesthetized with urethane presented respiratory- and pulse-related signals. In ten of these rats, the severity of these signals prevented juxtacellular stimulation. In contrast, in nine out of 10 animals anesthetized with 3-C, juxtacellular stimulation was feasible. Of these nine animals, the respiration- and pulse-related signals were absent in three, transient and if present only weak in one, and moderate in the remaining five animals. The remaining animal presented strong artifacts, comparable to those under urethane, and juxtacellular stimulation was impossible.

#### 2.2.2. Considerations on Statistical Power

**Figure 3:**
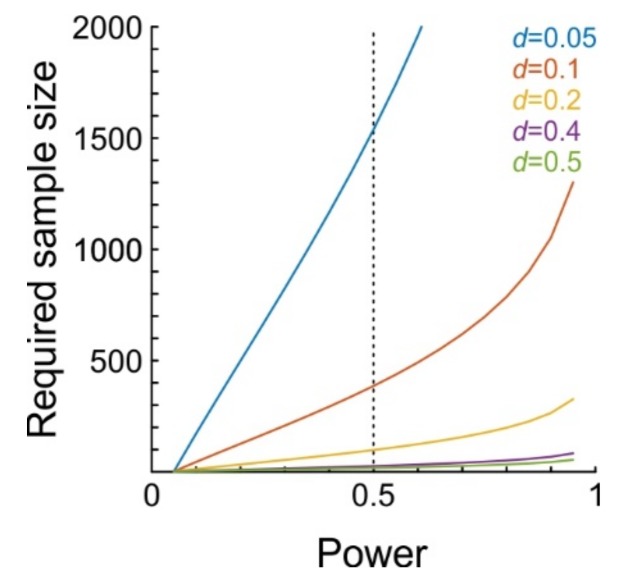
Results from the a-priori power analysis. Plot shows required sample size (ordinate) as a function of desired statistical power (abscissa) for five different effect sizes *d_z_* (color-coded). Vertical dotted line represents a power of 0.5.

The stability of the juxtacellular configuration is an indispensable methodological component. Although single neurons are certainly able to influence the activity of the local neuronal network, the effects are expected to be extremely small in the vast majority of cases. In order to detect such subtle effects, it will be crucial to achieve a large number of stimulation trials. To obtain a rough estimate as to how many trials are required, we performed a-priori power analyses for *t*-tests on dependent samples. Figure 3 shows the number of trials required to achieve a certain level of statistical power. Power denotes the probability of detecting a certain effect (i.e., yielding a significant test result), assuming the effect really exists and has a certain magnitude, where magnitude is usually calculated as Cohen’s *d_z_* (Methods). We calculated power for effect sizes ranging from *d* = 0.05 to *d* = 0.5 (*d* = 0.2 and *d* = 0.5 are considered small and moderate effect sizes, respectively [32]). For example, to achieve a statistical power of 0.5 (i.e., detecting 50% of network units which are affected by single-cell stimulation) at an alpha level of 0.05, 98 trials are needed when *d* = 0.2, but 386 when *d* = 0.1, and 1538 trials when *d* = 0.05.

Another analysis to detect significant effects on neuronal activity is to not look at individual network units (conducting one hypothesis test per cell, where mean firing rates before and during stimulation are compared across trials), but to compute statistics across units (calculate mean firing difference between baseline and stimulation epochs for each unit, and then conduct a single hypothesis test across all recorded units). In fact, the latter strategy was followed in the single-cell stimulation papers by others [24,25,33], because only low numbers of trials could be run in behaving animals. With this approach, the rationale is to still obtain as many trials as possible (to precisely estimate mean firing rate differences, as the precision of the estimate increases with the square root of the trial number). Importantly, the results of the power analysis also apply in this situation, although the interpretation of *d_z_* differs (applying to effect size for individual cells versus applying to the average effect size across a sample of cells). Thus, regardless of the statistical analysis approach, we would still want to have a large number of trials per cell, as well as a large number of cells.

Due to the paucity of previous research, as well as due to the varying readouts of single-cell stimulation effects (e.g., movement, detection performance, change in brain state), it is difficult to decide which effect size to expect (above and beyond “as many trials/cells as possible”). Taking the 5% increase in detectability reported in [21] to translate to a 5% increase or decrease in neuronal activity (of a single unit or the averaged network activity), we obtain an effect size of *d_z_* = 0.125, demanding for roughly 400 trials/cells to achieve a statistical power of 0.5. Taking the 0.06 Hz increase in firing rate (relative to 1 Hz) detected for evoking only a single spike per trial [34] and positing Poisson-distributed spike counts, we obtain an effect size of *d_z_* = 0.07, for which 786 trials would be needed to achieve a power of 0.5 (still implying 50% false negatives). Thus, given a trial duration of 2.5 s (yielding 24 trials per minute), the necessity of achieving stable juxtacellular configurations for tens of minutes to increase the number of stimulation trials becomes evident.

#### 2.2.3. Example Results from Juxtacellular Stimulation

To demonstrate the feasibility and showcase the data structure obtained with the aforementioned method, we analyzed electrophysiological data from two animals, JR16 and JR20 anesthetized with 3-C. The stimulated individual neurons (*n* = 17) were located at an average depth of 1300 µm (range: 443–1853 µm, JR16: 845–1853 µm (vertical penetration), JR20: 443–1757 µm (penetration perpendicular to cortical surface)) below the dura mater.

The juxtaposed neurons were stimulated with 200 ms square current pulses flanked by 10 ms rising and decay phases and delivered at 0.2 (JR16) or 0.5 Hz (JR20). We only considered stimulus trials that produced at least one action potential more during the 220 ms stimulus period, compared to baseline. On average, this led to an exclusion of 34 trials (range: 0–103) resulting in the presentation of on average 545 stimuli (range: 74–1263, JR16: 74–634, JR20: 518–1263 stimuli) per juxtacellular recording. The average current amplitude applied during each juxtacellular configuration was 5.2 nA (range: 1.9–14.5).

**Figure 4:**
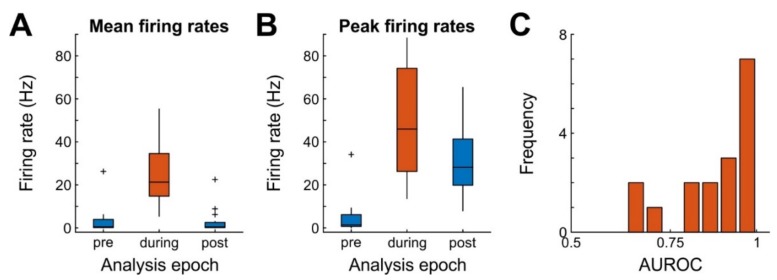
Efficacy of nanostimulation for 17 cells. (**A**) Mean firing frequencies of juxtaposed neurons in a 220 ms long time window each before, during and after the application of a 220 ms current pulse (analysis epoch). Box plots depict the median (middle horizontal line), data within the 25th–75th percentile (colored boxes), data within 1.5 times the interquartile range (whiskers), and individual outliers (+) (**B**) Same as A, but for peak firing frequencies of the same neurons, computed over 5-ms bins. (**C**) Histogram of nanostimulation effect sizes, quantified as the area under the receiver-operating characteristic (AUROC) curve. AUROC = 0.5 denotes complete overlap of two distributions (here: spike count distribution during 220 ms before stimulation and spike count distribution during 220 ms stimulation), AUROC = 1 denotes complete separation (i.e., all spike counts during stimulation were higher than the highest spike count before stimulation).

The juxtacellular units (JR16: *n* = 8, JR20: *n* = 9) had an average amplitude of 4.3 mV (range: 1.6–12.1). The average firing frequency immediately before, during, and after the stimulus (each 220 ms intervals) was 3.1 Hz (range: 0–26.2 Hz), 24.4 Hz (range: 5.4–55.3 Hz), and 2.9 Hz (range: 0–22.5 Hz), respectively. Firing frequencies were not equal across time (repeated measures ANOVA, *n* = 17, F(1,16) = 53.6, *p* = 0.003, Figure 4A). Similar results were obtained for peak firing rates (reaching 5, 50, and 30.4 Hz before, during, and after stimulation respectively; F(1,16) = 34.2, *p* < 0.001; Figure 4B). These data demonstrate that the stimulus was adequate for forcing juxtaposed neurons to generate bursts of action potentials with moderate to high effect sizes (Figure 4C).

**Figure 5:**
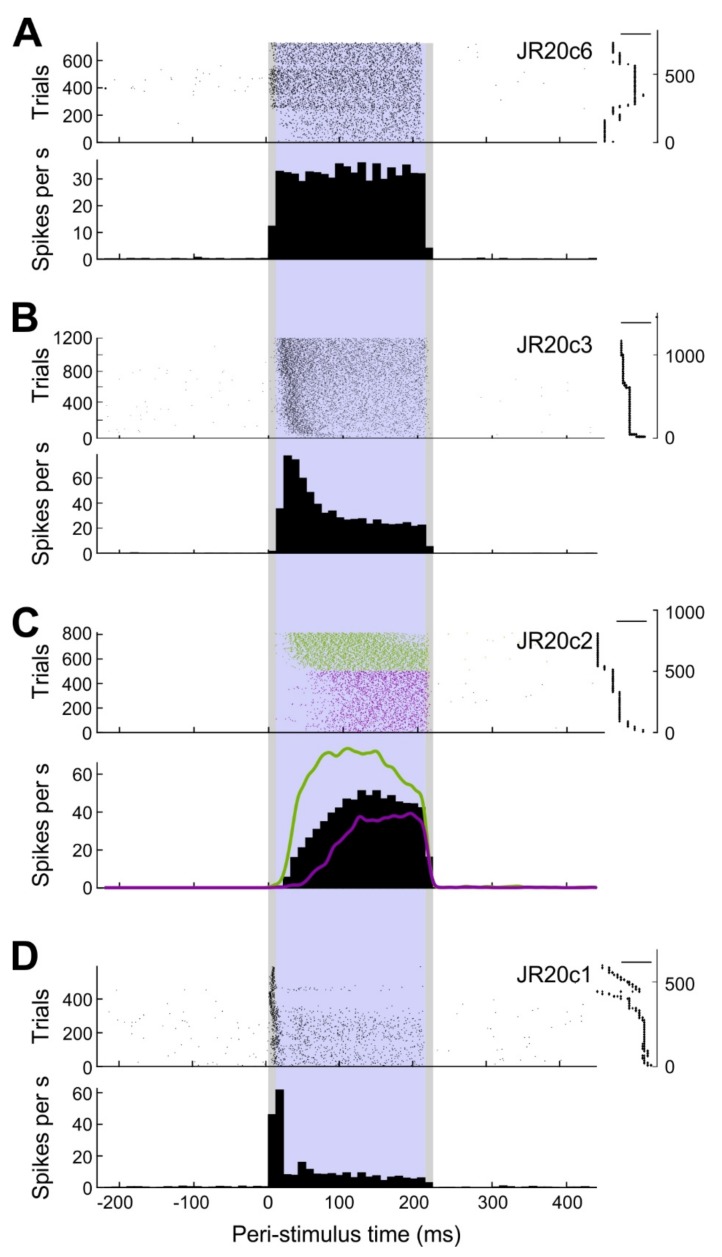
Types of responses of neurons to juxtacellular stimulation: (**A**) Steady firing cell. Top: raster plot with trials sorted according to their occurrence. Right: stimulus amplitude over time corresponding to the trials in the raster plot. Scale bar: 2 nA. Bottom: peri-stimulus time histogram (PSTH) with 10 ms bins. Grey shaded area: 10 ms rise and fall phase of the stimulus. Purple shaded area: plateau phase of the stimulus. (**B**) Same as A, but for a descending firing cell. Scale bar: 10 nA. (**C**) Same as A, but for an ascending firing cell. Scale bar: 4 nA. Subsets of the data are highlighted in green and magenta. Spike density functions (Gauss, 5 ms) overlaid in the PSTH. (**D**) Same as A, but for a bursting cell. Scale bar: 5 nA.

The average firing frequency during stimulation was assessed as a coarse measure of cellular response. Basically, response patterns could be classified into three categories (Figure 5). Throughout stimulation, cells either showed a steady (*n* = 10), decelerating (*n* = 6), or accelerating (*n* = 1) response, as determined by the significance and sign of the slope in a linear regression analysis based on 10-ms bins of firing rate during the stimulation epoch.

These results indicate that a square current stimulus can elicit distinct response patterns. However, response type might also change with different stimulus amplitudes. The exemplary analysis depicted in Figure 3C shows a slowly ascending response during earlier stimulus application but a steeper response during later stimulus applications. The cell was stimulated with an average of 4.5 nA during the first 500 trials and with 5.9 nA during the remaining 314 trials. A similar trend was seen in eight other cells. These observations suggest a negative correlation between the stimulus amplitude and the latency of spike generation following stimulus onset; however, we have not yet systematically investigated this issue.

### 2.3. Impact of Juxtacellular Stimulation on Local Network Activity

To assess the impact of the juxtacellular stimulation onto the local network, the neuronal network activity related to 17 juxtacellular configurations was analyzed. In JR16, the silicon probe penetrated the brain near-perpendicular to the cortical surface and the pipette penetrated the brain vertically. In JR20, both the silicon probe and the pipette penetrated the brain near-perpendicular to the cortical surface and were each rotated about 15° towards one another. The cortical penetration site of the silicon probe was on average located 568 µm (range: 286–1210 µm, JR16: 511–1210 µm, JR20: 286–329 µm) away from the cortical penetration site of the glass pipette. The trajectories of the silicon probe and glass pipette paths converged with advancing depth for JR20, but not for JR16. These data indicate that, in order to achieve a distance of a few hundred µm between the pipette and silicon probe penetration sites, it is of crucial importance to be able to incline and rotate not only the micromanipulator holding the silicon probe but also the micromanipulator holding the pipette.

In total, 630 local network units (JR16: 256 units, JR20: 374 units) were identified. To remain within the scope of describing the establishment of the method and present a preliminary data set to demonstrate the feasibility of the experimental approach, we did not further classify the network units. However, with a more comprehensive data set, we expect specifics of local network units, e.g., putative excitatory versus putative inhibitory units or resident cortical layer, to affect the transmission efficacy of the juxtacellular stimulation.

**Figure 6:**
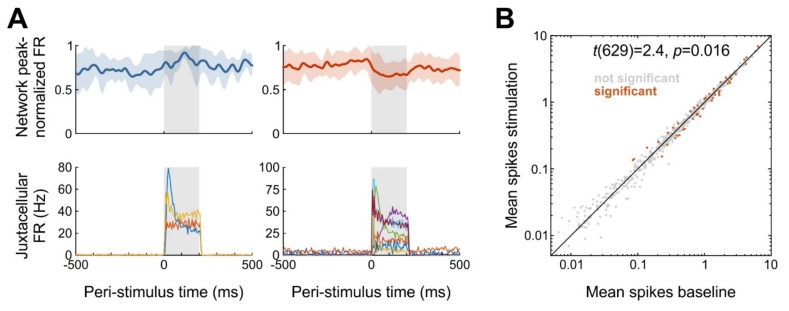
Impact of single-cell stimulation on local network activity. (**A**) Top: averaged peak-normalized spike-density functions (SDFs) of five (**left**) and 14 (**right**) network units with the lowest *p*-values; shadings represent standard deviation. SDFs were constructed by convolving peri-stimulus time histograms with 1-ms bins by an exponentially modified Gaussian kernel with time constant and standard deviation both set to 10 ms. Juxtacellular stimulation epoch is indicated by gray background. FR, firing rate. Bottom: peri-stimulus time histograms of three (**left**) and seven (**right**) single neurons stimulated in the juxtacellular configuration, pertaining to the network units in the respective top panels. Network units were selected on the basis that 15 and 16 significant units were expected to increase and decrease firing by chance (i.e., false positives) according to our bootstrap analysis, while 20 and 30 units were actually observed to do so. If we plotted all significant units (as is commonly performed in similar analyses), we would expect 75% (15 of 20; increasing) or 50% (16 of 30; decreasing) of the units to be false positive detections and actually not modulated. To somewhat decrease these percentages, we arbitrarily chose to sort units by *p*-value and then to select the 20 − 15 = 5 and 30 − 16 = 14 units with the lowest values. (**B**) Across-unit comparison for 630 network units recorded from two animals in which a total of 17 cells were stimulated in the juxtacellular configuration highlighting network units with significant firing rate changes.

As a first step toward answering our central research question if and how single-cell stimulation affects local network activity, we calculated firing rates of network units during and before single-cell stimulation in 200-ms windows. As expected, the majority of local network units was unaffected. However, we observed 50 out of 630 network units (8 %) with a significant change in mean firing rate during the 200-ms stimulation window, compared to firing rates immediately before stimulation (paired t-tests). Of these 50 units, 20 increased and 30 decreased firing rate (Figure 6A). With a false-positive level of 5% 31.5 units are expected to turn out significant if single-cell stimulation is ineffectual, and the probability of observing 50 or more significant units is less than 0.002 (binomial test). As a further test, we bootstrapped the distribution of expected numbers of significant units, using randomly selected 200-ms windows throughout the whole durations of recordings (1000 iterations). We found that the 95% confidence interval for the numbers of units significantly increasing and decreasing firing rates were both [9,24] (medians 15 and 16, respectively), confirming that more units than expected changed firing rate during stimulation. Lastly, we compared mean firing rates before and during stimulation (i.e., calculating statistics across all network units) and found that the firing rate during stimulation was significantly decreased by 0.05 Hz (*t*(629) = 2.4, *p* = 0.016; Figure 6B). Together, all three analyses demonstrate that 1) more network units than expected by chance change their activity level during single-cell stimulation and 2) that the average effect of single-cell stimulation on the local network is to decrease activity levels, at least on the time scale (200-ms) investigated here.

Having established that single-cell stimulation subtly but significantly affects network activity, we next asked whether certain parameters of the stimulation influenced the effect on the network. Specifically, we regressed effect size (firing rate changes of each network unit) on the distance to the stimulated cell (estimated through the distance between probe and pipette insertion sites) and the induced firing rate of the stimulated cell. To examine the effect of distance, we split the data into “close” and “far”, applying a threshold of 500 µm. As expected, we found that distance mattered: 39 out of 374 ‘close’ units (10%) turned out significant, with 15 and 24 units increasing and decreasing firing rate, respectively (expected number is 9.35 for either). In contrast, only 10 out of 256 “far” units (4%) turned out significant, similar to what would be expected by chance (12.8 units, 5%).

In view of the small effect sizes together with the small sample size (17 stimulated neurons), one cannot expect to identify relationships between stimulation parameters (number of induced spikes, coefficient of variation etc.) and the response of the network units. Still, we asked whether the effect size of the juxtacellular stimulation and the network response were significantly correlated, which was not the case (*r* = −0.01, *p* = 0.81).

## 3. Discussion

Here, we describe the feasibility of a technical approach to measure the activity of neurons across all layers of cerebral cortex while driving a single nearby neuron to fire high-frequency bursts of action potentials. The development of this technique was motivated by a growing body of evidence demonstrating that single cortical neurons can have a marked impact on a variety of parameters, including brain state, movement, and sensation, while, at the same time, the network mechanisms underlying these effects are mostly unknown [35]. Since effect sizes in previous studies were rather small, we performed a power analysis to get an impression as to how many trials (and network units) will be necessary to detect single-cell effects with reasonable likelihood. The analysis showed that on the order of 400 trials/cells will likely be necessary to reliably observe single-cell induced network modulation, and therefore a highly stable and reliable stimulation-recording configuration is required. Building on previous work [22,30,36], we demonstrate how such a configuration can be achieved, and we present pilot data indicating that significant, albeit small effect sizes can be detected.

We are employing the setup to test the hypothesis that single-neuron activation in cortex in vivo exerts measurable effects on the local neuronal network, possibly due to recurring excitation [37]. To our knowledge, this has only been attempted a few times before (but see, e.g., [38,39] for related in vitro work). In urethane-anesthetized rats, Li and Dan [40] used whole-cell patch-clamp recordings to electrically induce prolonged (~5 min duration) high-frequency bursting activity in pyramidal neurons in supragranular layers and found that, for roughly half of the stimulated neurons, local field potential activity patterns several millimeters away from the patched neurons could be altered. In another elegant study, Kwan and Dan [41] combined two-photon imaging of superficial layers of visual cortex with electrical stimulation of single neurons to examine the effects of single-cell bursting on nearby neurons, dissecting the role of different subtypes of neurons.

In all single-cell stimulation studies discussed thus far, multiple action potentials were induced at high frequencies. Choosing a different approach, London et al. [34] investigated whether the introduction of a single action potential into an individual cortical neuron in layer 5 causes a measurable change in the network activity. Similar to our approach, multi-unit activity was recorded with a 16-channel multi-electrode array positioned in close proximity (< 300 µm) of the cortical neuron, which was electrically stimulated with a patch electrode. Their finding that a single spike added to the network yields measureable effects is perhaps the strongest demonstration of the power of single neurons to influence overall cortical processing.

Although we did find a significant effect of single-cell stimulation in our preliminary data set, the fraction of affected units (50/630 or 8%, compared to chance expectancy of 5%) as well as the average effect sizes were small and therefore on the verge of detectability. Dissecting the effects of single-cell activation on the network in vivo, as has frequently been done in vitro [42,43,44], will require fine-tuning of the experimental approach in at least two regards: (1) more extensive characterization of input-output functions, e.g., by modifying stimulation parameters, and (2) performing more fine-grained data analysis on much larger sets of stimulated cells.

At present, little is known about how evoked spike patterns relate to network effects. Stimulating in motor cortex, Brecht et al. [19] found larger movement amplitudes for higher spike frequencies. In contrast, Doron et al. [24] found a negative correlation between spike frequency and the magnitude of the perceptual bias, and a more pronounced effect for irregular as compared to regular spike trains. Especially the latter effect is highly interesting because it suggests that the network is highly sensitive to patterned stimulation.

The elevated effectiveness of irregular over regular patterns could be explained on the basis of in vitro studies. Central synapses are characterized by low transmitter release probability (~0.2–0.3) and are therefore notoriously unreliable [45,46]. However, when two consecutive action potentials reach a presynaptic terminal within a short latency, the release probability may increase dramatically (short-term facilitation; [47]). Synapses of that kind would only be unreliable in the sense of ineffective transmission of individual action potentials, whereas bursts with two or more spikes may consistently result in transmitter release and postsynaptic spiking. Accordingly, such synapses serve as high-pass filters. Further elaborating this notion, Izhikevic et al. [48] postulated that individual synapses function as band-pass filters. In this scenario, synaptic facilitation and depression operate at the same synapse, but affect different frequency bands. Thus, synaptic depression provides low-pass filtering while synaptic facilitation implements high-pass filtering, effectively resulting in the band-pass filtering of incoming spike trains. Low-pass filtering may explain the aforementioned findings that the animals’ detectability of single cell stimulation was negatively correlated with the induced action potential frequency [24], while irregular spike patterns (composed of high-frequency bursts with variable inter-burst intervals) might have tapped the pass-band’s sweet spot.

To answer the question which temporal patterns exert strong and weak effects onto the local network, a method is required by which arbitrary action potential sequences can be evoked in individual cortical neurons. It has been demonstrated multiple times that nanostimulation can be used to parametrically vary both spike number and spike frequency ([24,30]; also see Figure 5). Recently, we developed a current stimulation protocol which enables the evocation of a specific number of action potentials at a defined frequency, or an arbitrary temporal pattern [49]. This technique, dubbed “kurzpuls-nanostimulation” (KP-nanostimulation), uses short (~7 ms) pulses of a few nanoampere to induce action potentials. In contrast to square pulses, which are used for classical nanostimulation, kurzpulses do not produce artifacts in the simultaneously recorded band-pass filtered voltage signal of the same electrode, such that both evoked and spontaneous action potentials can be detected during and outside stimulation epochs. Experiments in which the number, regularity, and frequency of evoked action potentials are systematically varied in order to examine which stimulation patterns are most effective are currently ongoing.

In this proof of principle study, we compared spike counts in and outside of stimulation windows, collapsing all juxtacellularly stimulated neurons. With larger samples, it will be possible to (1) separately analyze the effects of neurons in different layers and with different stimulation profiles (e.g., steady versus burst firing, Figure 5), (2) separately analyze the effects within and across different cortical layers, and (3) separating putative excitatory (broad-spike) and inhibitory (thin-spike) neurons [24,25].

The finding that the activation of a single neuron provokes a change in global brain state, movement, and sensation, raises a multitude of questions. How do evoked signals propagate across different cell types in different cortical layers? Which cell types or populations are most crucial for mediating the effect of single-cell stimulation? How are mediating neurons embedded in the local neuronal network? Do long-term potentiation and depression play a role? We are confident that the approach presented in this paper, especially when combined with other techniques such as barrel column localization through intrinsic optical imaging or electrocorticography, along with histological verification of cell type, will be helpful in answering these questions.

The in vivo experimental approach described in this feasibility study can in principle be applied to any desired brain region to address the question of single-cell effects onto local networks, which likely differs between areas of different cytoarchitectonic structure. Furthermore, it could be used to investigate the processing of bottom-up sensory information, e.g., by stimulating in the trigeminal ganglion and recording in the trigeminal brain stem complex or thalamus. Another application would be to investigate local signal processing while activating or silencing specific neuronal populations or neuromodulatory inputs optogenetically or pharmacologically.

## 4. Materials and Methods

### 4.1. Ethical Approval and Animals

All procedures were approved by local authorities (Landesuntersuchungsamt Rheinland-Pfalz) and in accordance with the European Communities Council Directive of September 22nd, 2010 (2010/63/EU). Furthermore, the principles of laboratory animal care and use were followed (National Research Council, 2011).

21 rats of different strains (1 Long Evans (Janvier), nine Sprague Dawley (Janvier, Saint-Berthevin Cedex, France) and 11 Wistar Han (Charles River, Sulzfeld, Germany)), were used. On average, animals had an age of 10 weeks (min: 7, max: 13) and a weight of 320 g (min: 125, max: 511) at the time of the experiment.

### 4.2. Preparation

#### 4.2.1. Anesthesia and Surgical Preparation

Anesthesia was induced with 5% isoflurane in 100% oxygen, supplied via an anesthesia system (EZ-108SA, Euthanex Corp., Palmer, PA, USA). Upon loss of the righting reflex, isoflurane was reduced to 2.5%. To allow a smooth induction, it proved vital to habituate the animals to the acrylic plastic induction chamber. Cereals were used to form positive associations with the induction chamber and as distractors during induction.

Once the animals were unresponsive and reached a stable slow breath rate, they were injected intraperitoneally (i.p.) with the anesthetic that was used throughout the remainder of the experiment. Eleven animals received 1.5 g urethane per 1 kg body weight (BW). Two of these animals received additional subcutaneous (s.c.) injections of 0.05 or 0.11 mg atropine/kg BW. In the other 10 animals, a three-component anesthetic (3-C, in mg/kg BW, 0.15 Medetomidine, 2.00 Midazolam, 0.005 Fentanyl) was administered i.p. Depth of anesthesia was judged by assessing breath rate, whisker twitching, as well as palpebral and toe pinch reflexes. All animals were supplemented with the respective anesthetic as needed to ensure stage-3 anesthesia.

Trimming fur on the head and whiskers was commenced upon the absence of the palpebral reflex. The animals were subsequently placed on a paper towel covered thermal pad, body temperature was measured by a rectal probe and kept at 37 ± 1 °C by a small animal temperature controller (ATC-2000, WPI, Sarasota, FL, USA).

When the toe-pinch reflex was absent, the animal was fixed to the stereotactic frame (parallel rail base, Stoelting, Dublin, Ireland) and the skin was treated with lidocaine hydrochloride. An incision was made and the skull was exposed and cleaned. The breath rate was monitored throughout the experiment via a piezo-ceramic transducer positioned underneath the chest [50] and connected to a data acquisition unit (1401 Power3A, Cambridge Electronic Design (CED), Cambridge, UK). When positioned ideally, heart rate signals were picked up as well. The majority of animals under 3-C anesthesia received subcutaneous (s.c.) injections of 0.9% NaCl and 5% Glucose to avoid dehydration and hypoglycemia.

#### 4.2.2. Surgical Site

The surgical site was illuminated with a cold light source (KL 1600 LED, Schott, Mainz, Germany), visualized with a stereomicroscope (M80, Leica, Wetzlar, Germany) and displayed via a color camera (DFK MKU130-10x22, The ImagingSource, Bremen, Germany). To allow whisker stimulation, the animal’s head had to be freed from the stereotaxic frame. Therefore, a custom made head post was fixed to the skull. Two miniature stainless steel screws were screwed into drilled holes of the contralateral skull plate. A polyamide machine screw with a tightly fastened nut was placed up-side-down between these stainless steel screws. Dental cement was used to fix this arrangement in place. After completion of the craniotomy, the polyamide screw was inserted in a fitting frame and tightly fixed with a second nut above. This frame was attached to a heavy pole. In experiments without whisker stimulation, the animal remained fixed in the stereotactic frame.

The location of the craniotomy (1 to 4 mm posterior of bregma and 4 mm lateral of bregma until about 1 mm below the lateral ridge) was marked before the application of dental cement. Upon completion of the head post, the craniotomy was created with a dental unit polisher whilst the dura mater was kept intact. Thereafter, a chlorided silver ball grounding wire was fixed to the skull using dental cement such that the ball was positioned ventral of the craniotomy. Anterior and posterior of the craniotomy, a coronally sloping wall was created with dental cement to support the retention of saline to keep the brain moist.

Agar (1–3% *w*/*v*) was applied onto the brain of five animals anesthetized with urethane. The presence of agar did not prevent respiratory signals at the glass pipette. However, agar impeded the detection when the pipette started penetrating the brain, such that depth readings of the micromanipulator were worthless and the depth of the pipette could not be estimated online. The brains of the remaining animals were covered with Ringer solution only.

### 4.3. Probe Positioning and Penetration

#### 4.3.1. Single Cell Stimulation

Glass pipettes were pulled from borosilicate glass capillaries (GB150F-8P, Science Products, Hofheim, Germany) using a horizontal puller (P-1000, Sutter Instruments, Novato, CA, USA) and utilized when they had a resistance of 3–8 MΩ, whilst care was taken to create a long and slim taper to minimize the tissue displacement when the tip was advanced to infragranular cortical layers.

Glass pipettes were mounted on a microelectrode holder with side port (PPH-1P-BNC-0-1.5, npi electronic, Tamm, Germany), attached to a head stage and connected to an amplifier (ELC-03XS, npi electronic) which allowed simultaneous recording and stimulation. While advancing through the brain a pressure of approximately 20 hPa (assessed by a manometer) was applied and adjusted when necessary. Stimulation protocols were applied via custom-written protocols in Spike 2 and triggered via a data acquisition unit (1401 Power3A, CED). Signals were low-pass filtered at 3 kHz, digitized at 20 kHz and displayed in Spike2 (version 8.11, CED). Spike sorting was conducted offline in Spike 2.

The distance between the penetration sites of the glass pipette and the silicon probes was measured either by moving the pipette, after completion of cell stimulation and retraction from the cortex, from its penetration site to the penetration site of the silicon probe and recording the covered distance in the *x*- and *y*-axis, or by using the calibrated measure function in the IC Measure software of a microscope mountable color camera (DKF MKU130-10x22, The Imaging Source).

Initially (incl. JR16), the head stage was mounted on a motorized micromanipulator that could neither be tilted nor rotated (IVM-3000, Scientifica, Uckfield, UK). Hence, the pipette penetrated the brain vertically and depth recordings of the micromanipulator do not correspond to cortical depth. In seven animals used for this feasibility study and all following experiments, the head stage was mounted on a four-axis motorized micromanipulator (Junior, Luigs & Neumann, Ratingen, Germany). The small dimensions of the micromanipulator, combined with the ability to tilt the one axis and rotate the base, proved vital to enable positioning of the pipette in close proximity to the silicon probe.

For post-hoc identification of stimulated neurons, pipettes were filled with an biocytin-enriched intracellular solution containing (in mM/L) 126 K-gluconate, 10 HEPES, 20 KCl, 0.5 EGTA, 10 Na_2_-phosphocreatine, 4 MgATP, 0.3 Na_3_GTP, and 1.5% (*w*/*v*) biocytin, adjusted to a pH of 7.2 with 5 M KOH. No specific staining pulse was applied to transfer biocytin into the cell [23]. Instead, the experimental stimulus protocols (square pulse, 200 ms, 1 to 20 nA, 0.2 Hz) proved sufficient for staining neurons. After completion of a recording, the pipette was slowly retracted to leave the cell unharmed. If a cell was not intended to be recovered the pipette was advanced deeper. Nonetheless, a precise record of the location of any encountered and stimulated neurons was kept to allow post-hoc identification.

#### 4.3.2. Local Network Recording

Two kinds of silicon probes (NeuroNexus, Ann Arbor, MI, USA) were used for recording local network activity. In both cases, 32 channels were recorded and contacts on the shanks were placed linearly. In one probe, all channels were positioned on a single shank with contacts spaced 50 µm apart (JR16 and JR20). In the other probe, the 100 µm spaced contacts were distributed on two shanks which were separated from each other by 500 µm. Signals were transferred to a data acquisition system (AlphaLab SNR, AlphaOmega, Nazareth Illit, Israel) and continuously sampled at 22 kHz. Spike sorting was conducted using Offline Sorter v4 (Plexon, Dallas, TX, USA).

Probes were mounted on a motorized micromanipulator (S-IVM-1000, Scientifica), which was mounted on a manual micromanipulator (ultra-precise manipulator arm, Stoelting). The manual micromanipulator could be tilted in its vertical axis and rotated on its base which was critical for positioning the silicon probe such that a glass pipette could be placed in close proximity.

For post-hoc identification of silicon probe location, the shanks were coated with DiI (42364, Sigma Aldrich, Taufkirchen, Germany) by repeatedly dipping in 70% ethanol saturated with DiI prior to penetration of the brain. Reliable recovery was obtained with up to two consecutive penetrations.

### 4.4. Data Analysis

We analyzed the electrophysiological data from two animals (Wistar Han; JR16: 91 days, 380 g, JR20: 61 days; 125 g).

The voltage traces of the juxtacellular recordings were processed in Spike 2 and band-pass filtered from 300 to 3000 Hz (finite impulse response filter, transition gap: 200 Hz). Spikes were extracted with amplitude thresholds at 0.5 mV or higher (noise band usually ~±0.15 mV). Due to the outstanding signal-to-noise ratio owing to the juxtacellular configuration, no further spike sorting techniques were applied, unless further units of different amplitude were readily identifiable. The spiking activity was assessed in a 220 ms analysis window starting at the onset of the stimulus until the offset of the stimulus, including the 10 ms rise and fall phases. The activity of the stimulus was compared to the activity during a baseline of 220 ms, directly preceding the stimulus onset. Post-stimulus activity was assessed in a 220 ms analysis window directly following the stimulus offset.

The voltage traces of local network recordings were processed in Offline Sorter and high-pass filtered at 500 Hz (2-pole Bessel). Events surpassing a threshold of four (JR16) or five (JR20) standard deviations below the noise band were considered putative spikes. Semi-automated sorting strategies were applied to separate distinct single- and multi-units, which were collapsed for the analyses presented in this paper.

To exclude potential stimulus onset or offset artifacts, we moved the analysis window by 5 ms into the rise phase of the juxtacellular stimulation. To assess the effect of juxtacellular stimulation on network activity, we performed three different analyses: first, we counted spikes both during the stimulation window as well as during a 200-ms window immediately preceding stimulation, and performed paired t-tests for each network unit. The resulting number of units with significant modulation was then compared to the expected number using a binomial test. Second, we performed a bootstrapping analysis, in which we selected random time points during the recording and then compared spike counts in the 200 ms before to those during 200 ms after those time points, and performed paired t-tests. This procedure was repeated 1000 times to derive a distribution of the numbers of significant units (which were entirely false positives), and compared the resulting bootstrapped distribution to the actually observed numbers of significant units. Third, we calculated mean firing rates during baseline and stimulation for each neuron, and performed a single paired t-test across all network units.

Power analyses were performed using G*Power 3.1 [51]. Effect size *d_z_* was calculated as:

$$d_{z}=\frac{\left|m_{x}-m_{y}\right|}{\sqrt{s_{x}^{2}+s_{x}^{2}-2r_{xy}s_{x}s_{y}}}$$

where *s_x_* and *s_y_* denote standard deviations of data vectors x and y, *m_x_* and *m_y_* denote sample means, and *r_xy_* denotes the Pearson product-moment correlation between *x* and *y*. All other analyses were performed using custom-written code [52] in Matlab R2018b (The Mathworks).

## Abbreviations

3-C	3-component anesthesia

## References

[1] Panzeri S., Harvey C.D., Piasini E., Latham P.E., Fellin T. (2017). Cracking the neural code for sensory perception by combining statistics, intervention and behavior. Neuron.

[2] Averbeck B.B., Latham P.E., Pouget A. (2006). Neural correlations, population coding and computation. Nat. Rev. Neurosci..

[3] Shadlen M.N., Newsome W.T. (1998). The variable discharge of cortical neurons: Implications for connectivity, computation, and information coding. J. Neurosci..

[4] Chapin J.K., Lin C.S. (1984). Mapping the body representation in the SI cortex of anesthetized and awake rats. J. Comp. Neurol..

[5] Reyes-Puerta V., Kim S., Sun J.-J., Imbrosci B., Kilb W., Luhmann H.J. (2015). High stimulus-related information in barrel cortex inhibitory interneurons. PLoS Comput. Biol..

[6] Ghazanfar A.A., Stambaugh C.R., Nicolelis M.A. (2000). Encoding of tactile stimulus location by somatosensory thalamocortical ensembles. J. Neurosci..

[7] Bair W., Koch C. (1996). Temporal precision of spike trains in extrastriate cortex of the behaving macaque monkey. Neural Comput..

[8] Britten K.H., Shadlen M.N., Newsome W.T., Movshon J.A. (1992). The analysis of visual motion: a comparison of neuronal and psychophysical performance. J. Neurosci..

[9] Stüttgen M.C., Schwarz C. (2008). Psychophysical and neurometric detection performance under stimulus uncertainty. Nat. Neurosci..

[10] Stüttgen M.C., Schwarz C. (2010). Integration of vibrotactile signals for whisker-related perception in rats is governed by short time constants: comparison of neurometric and psychometric detection performance. J. Neurosci..

[11] Zohary E., Shadlen M., Newsome W. (1994). Correlated neuronal discharge rate and its implications for psychophysical performance. Nature.

[12] Olshausen B.A., Field D.J. (2004). Sparse coding of sensory inputs. Curr. Opin. Neurobiol..

[13] Laughlin S.B. (2001). Energy as a constraint on the coding and processing of sensory information. Curr. Opin. Neurobiol..

[14] Lennie P. (2003). The cost of cortical computation. Curr. Biol..

[15] Butovas S., Schwarz C. (2003). Spatiotemporal effects of microstimulation in rat neocortex: A parametric study using multielectrode recordings. J. Neurophysiol..

[16] Butovas S., Schwarz C. (2007). Detection psychophysics of intracortical microstimulation in rat primary somatosensory cortex. Eur. J. Neurosci..

[17] Cohen M.R., Newsome W.T. (2004). What electrical microstimulation has revealed about the neural basis of cognition. Curr. Opin. Neurobiol..

[18] Histed M.H., Bonin V., Reid R.C. (2009). Direct activation of sparse, distributed populations of cortical neurons by electrical microstimulation. Neuron.

[19] Brecht M., Schneider M., Sakmann B., Margrie T.W. (2004). Whisker movements evoked by stimulation of single pyramidal cells in rat motor cortex. Nature.

[20] Herfst L.J., Brecht M. (2008). Whisker movements evoked by stimulation of single motor neurons in the facial nucleus of the rat. J. Neurophysiol..

[21] Houweling A.R., Brecht M. (2008). Behavioural report of single neuron stimulation in somatosensory cortex. Nature.

[22] Pinault D. (1994). Golgi-like labeling of a single neuron recorded extracellularly. Neurosci. Lett..

[23] Pinault D. (1996). A novel single-cell staining procedure performed in vivo under electrophysiological control: morpho-functional features of juxtacellularly labeled thalamic cells and other central neurons with biocytin or Neurobiotin. J. Neurosci. Methods.

[24] Doron G., von Heimendahl M., Schlattmann P., Houweling A.R., Brecht M. (2014). Spiking irregularity and frequency modulate the behavioral report of single-neuron stimulation. Neuron.

[25] Tanke N., Borst J.G.G., Houweling A.R. (2018). Single-cell stimulation in barrel cortex influences psychophysical detection performance. J. Neurosci..

[26] Herculano-Houzel S., Mota B., Lent R. (2006). Cellular scaling rules for rodent brains. Proc. Natl. Acad. Sci. USA.

[27] Schnepel P., Kumar A., Zohar M., Aertsen A., Boucsein C. (2015). Physiology and impact of horizontal connections in rat neocortex. Cereb. Cortex.

[28] Hellwig B. (2000). A quantitative analysis of the local connectivity between pyramidal neurons in layers 2/3 of the rat visual cortex. Biol. Cybern..

[29] Narayanan R.T., Egger R., Johnson A.S., Mansvelder H.D., Sakmann B., De Kock C.P.J., Oberlaender M. (2015). Beyond columnar organization: Cell type- and target layer-specific principles of horizontal axon projection patterns in rat vibrissal cortex. Cereb. Cortex.

[30] Houweling A.R., Doron G., Voigt B.C., Herfst L.J., Brecht M. (2010). Nanostimulation: manipulation of single neuron activity by juxtacellular current injection. J. Neurophysiol..

[31] Narayanan R.T., Mohan H., Broersen R., de Haan R., Pieneman A.W., de Kock C.P.J. (2014). Juxtasomal Biocytin Labeling to Study the Structure-function Relationship of Individual Cortical Neurons. J. Vis. Exp..

[32] Cohen J. (1992). A power primer. Psychol. Bull..

[33] Voigt B.C., Brecht M., Houweling A.R. (2008). Behavioral detectability of single-cell stimulation in the ventral posterior medial nucleus of the thalamus. J. Neurosci..

[34] London M., Roth A., Beeren L., Häusser M., Latham P.E. (2010). Sensitivity to perturbations in vivo implies high noise and suggests rate coding in cortex. Nature.

[35] Doron G., Brecht M. (2015). What single-cell stimulation has told us about neural coding. Philos. Trans. R. Soc. B-Biological Sci..

[36] Neto J.P., Lopes G., Frazão J., Nogueira J., Lacerda P., Baião P., Aarts A., Andrei A., Musa S., Fortunato E. (2016). Validating silicon polytrodes with paired juxtacellular recordings: method and dataset. J. Neurophysiol..

[37] Rodney J.D., Christof K., Misha M., Kevan A.C.M., Humbert H.S. (1995). Recurrent Excitation in Neocortical Circuits. Science.

[38] Miles R., Wong R.K. (1983). Single neurones can initiate synchronized population discharge in the hippocampus. Nature.

[39] Bonifazi P., Goldin M., Picardo M.A., Jorquera I., Cattani A., Bianconi G., Represa A., Ben-Ari Y., Cossart R. (2009). GABAergic hub neurons orchestrate synchrony in developing hippocampal networks. Science.

[40] Li C.-Y.T., Poo M.-M., Dan Y. (2009). Burst spiking of a single cortical neuron modifies global brain state. Science.

[41] Kwan A.C., Dan Y. (2012). Dissection of cortical microcircuits by single-neuron stimulation in vivo. Curr. Biol..

[42] Molnar G., Ola S., Komlosi G., Füle M., Szabadics J., Varga C., Barzo P., Tamas G. (2008). Complex Events Initiated by Individual Spikes in the Human Cerebral Cortex. PLoS Biol..

[43] Song S., Sjöström P.J., Reigl M., Nelson S., Chklovskii D.B. (2005). Highly nonrandom features of synaptic connectivity in local cortical circuits. PLoS Biol..

[44] Kapfer C., Glickfeld L.L., Atallah B.V., Scanziani M. (2007). Supralinear increase of recurrent inhibition during sparse activity in the somatosensory cortex. Nat. Neurosci..

[45] Allen C., Stevens C.F. (1994). An evaluation of causes for unreliability of synaptic transmission. Proc. Natl. Acad. Sci. USA.

[46] Volgushev M., Kudryashov I., Chistiakova M., Mukovski M., Niesmann J., Eysel U.T. (2004). Probability of transmitter release at neocortical synapses at different temperatures. J. Neurophysiol..

[47] Lisman J.E. (1997). Bursts as a unit of neural information: Making unreliable synapses reliable. Trends Neurosci..

[48] Izhikevich E.M., Desai N.S., Walcott E.C., Hoppensteadt F.C. (2003). Bursts as a unit of neural information: Selective communication via resonance. Trends Neurosci..

[49] Stüttgen M.C., Nonkes L.J., Geis H.-R.A., Tiesinga P.H., Houweling A.R. (2017). Temporally precise control of single neuron spiking by juxtacellular nanostimulation. J. Neurophysiol..

[50] Zehendner C.M., Luhmann H.J., Yang J.W. (2013). A Simple and Novel Method to Monitor Breathing and Heart Rate in Awake and Urethane-Anesthetized Newborn Rodents. PLoS ONE.

[51] Faul F., Erdfelder E., Lang A.-G., Buchner A. (2007). G*Power 3: A flexible statistical power analysis program for the social, behavioral, and biomedical sciences. Behav. Res. Methods.

[52] Hentschke H., Stüttgen M.C. (2011). Computation of measures of effect size for neuroscience data sets. Eur. J. Neurosci..

